# Supplementary material for: Hydrogenation of saturated organic and inorganic molecules in metallic hydrogen
Source: Nat Commun. 2025 Sep 29;16:8548. doi: 10.1038/s41467-025-63552-6 (PMC12479789; doi:10.1038/s41467-025-63552-6)
Supplement: Supplementary file 1 — Supplementary Information [file 41467_2025_63552_MOESM1_ESM.pdf]

Supplementary Information

Jakkapat Seeyangnok[1], Udomsilp Pinsook[1], and Graeme J Ackland[2]

[1]Department of Physics, Faculty of Science, Chulalongkorn University, Bangkok, Thailand.
[2]Centre for Science at Extreme Conditions, School of Physics and Astronomy, University of Edinburgh, Edinburgh, United Kingdom.

Structural substitution of carbon in metallic hydrogen

Supplementary Figure 1 shows the visualization of a structural substitution in metallic hydrogen, where one hydrogen atom is replaced by a carbon atom.

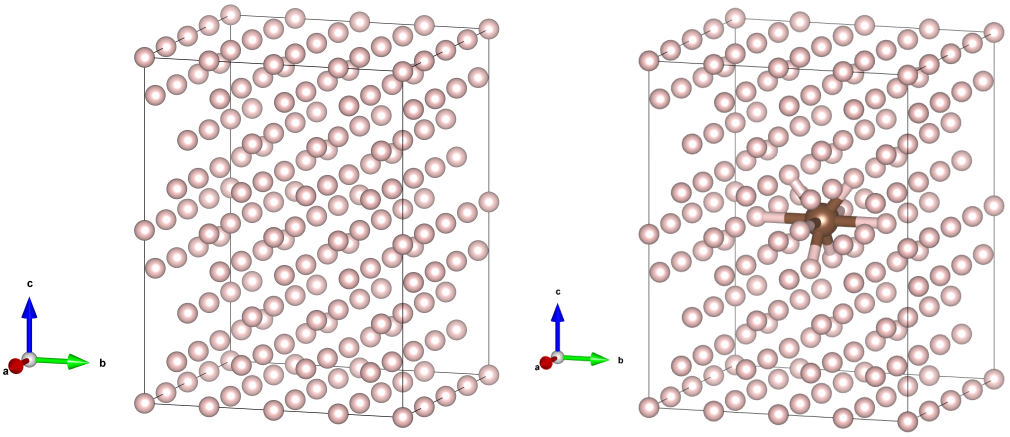

Supplementary Figure 1: show (Left) the supercell of 128 hydrogen atoms of the $I4_1/amd$ metallic structure and the structures of substitutional carbon when neighboring hydrogens are removed at different concentrations to investigate the solubility limit.

Free energy

We calculated the enthalpy (H), entropy contribution (TS), zero-point energy (U_{ZPE}), configurational entropy, and Gibbs free energy for various structural arrangements, with all values reported in eV per atom. Additionally, the free energy of solution (in eV) was evaluated for each configuration as shown in Supplementary Table 1.

Supercell	H_{MD}	H_{static}	TS (MD)	U_{ZPE} (MD)	G (MD)
CH_{126}	-10.792(1)	-10.91109	0.00988	0.32588	-10.476(1)
CH_{125}	-10.802(1)	-10.92137	0.00950	0.32125	-10.491(1)
CH_{124}	-10.814(1)	-10.93038	0.00875	0.32154	-10.501(1)
CH_{123}	-10.818(1)	-10.93814	0.01094	0.31637	-10.512(1)
C (diamond)	-144.085(2)	-144.35980	0.00414	0.27343	-143.815(2)
H (I4amd)	-9.759(1)	-9.87520	0.00488	0.33264	-9.432(1)
Supercell		H_{static}	TS (DFPT)	U_{ZPE} (DFPT)	
CH_{124}		-10.93038	0.00611	0.29304	
C (diamond)		-144.3598	0.00404	0.27565	
H (I4amd)		-9.87520	0.00358	0.29202	

Supplementary Table 1: shows thermodynamic properties for the solid solution at 300K and 500GPa, i.e. calculated enthalpy H, entropy TS, zero-point energy U_{ZPE}, configurational entropy, Gibbs free energy (all in eV/atom).

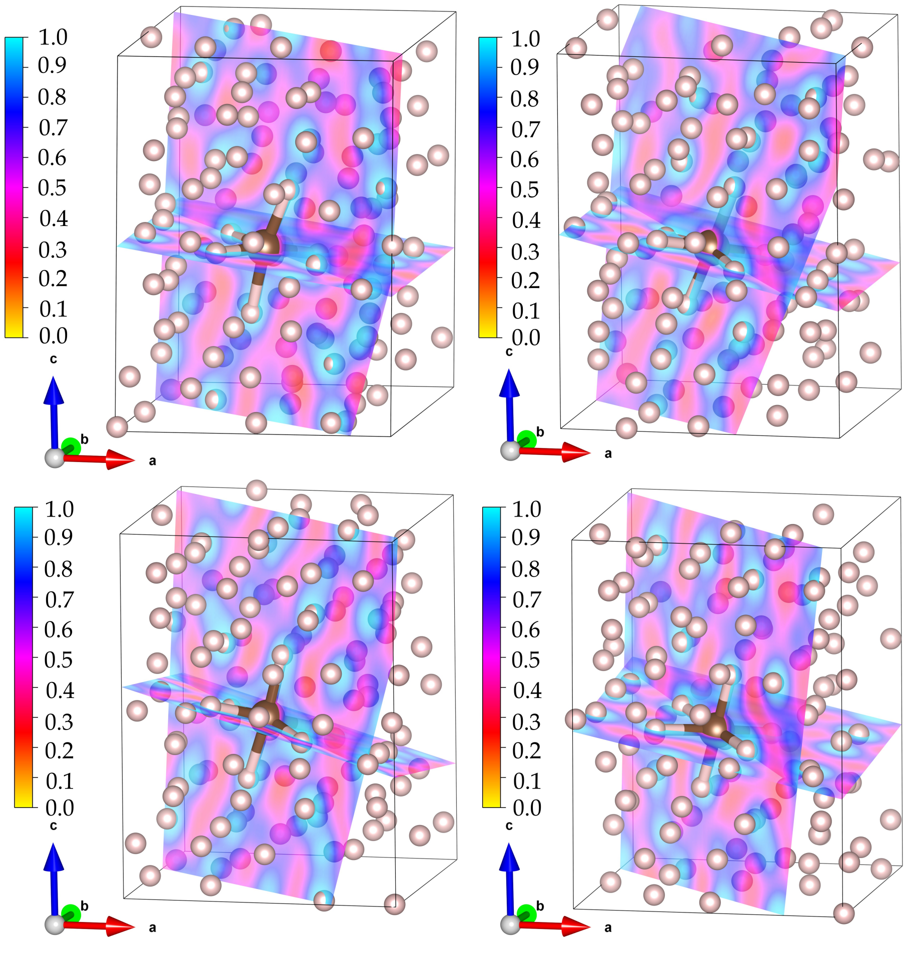

Supplementary Figure 2: show the Electron Localised Function (ELF) of CH124 for four different snapshots from CH_6 in solid metallic hydrogen.

Electron localization Function (ELF)

In this section, we show the electron localization function (ELF) of CH_6 in solid metallic hydrogen from four snapshots of CH_6 in Supplementary Figure 4 of CH124 at 300 K,

500GPa. All ELFs indicate high values between the carbon and the six surrounding hydrogen atoms as shown in Supplementary Figure 2. These ELFs also indicates that all of the six bonds are equivalent to each other.

Angle distribution

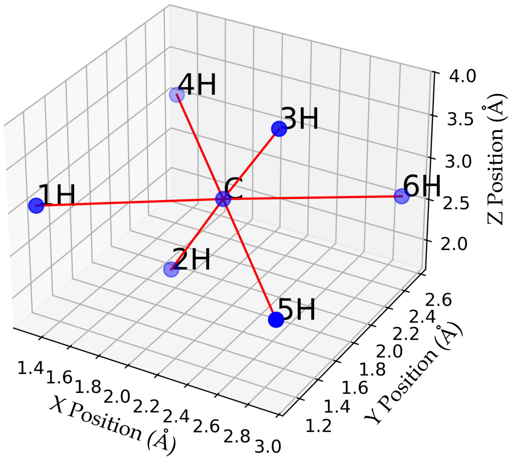

Supplementary Figure 3: show the average positions of CH_6 in solid metallic hydrogen at 300K where X, Y and Z are the positions of atoms in Angstorm.

The angle distribution of CH_6 in solid metallic hydrogen shows the angle distribution between pairs of hydrogen atoms with respect to carbon for each step of MD within the radius cutoff of 1.30Å where the average number of coordination is about 6 hydrogen atoms. In the case of solid metallic hydrogen, we also compute average positions of the six hydrogen atoms which turns out to be the same hydrogen throughout the MD simulation. The average position and the average angle are shown in Supplementary Figure 3 and Supplementary Table 2. Two significant peaks show probable angles at 90 and 180 degrees as shown in Supplementary Figure 4.

The angle distribution of CH_6 in liquid metallic hydrogen shows the angle distribution between pairs of hydrogen atoms with respect to carbon for each step of MD within the radius cutoff of 1.30Å where the average number of coordination is about six. Two significant peaks show probable angles at 90 and 180 degrees with large smearing of the distribution resulting from high anharmonicity of hydrogen as shown in Supplementary Figure 4.

The angle distribution of C_2H_8 in liquid metallic hydrogen shows the angle distribution between pairs of hydrogen atoms with respect to each carbon of C_1 and C_2 seperately for each step of MD within the radius cutoff of 1.30Å where the average number of coordination for each carbon is about four. Two significant peaks show probable angles at 75 and 144 degrees as shown in Supplementary Figure 4.

The angle distribution of C_3H_{10} in liquid metallic hydrogen shows the angle distribution between pairs of hydrogen atoms with respect to each carbon of C_1, C_2, and C_3 seperately for each step of MD within the radius cutoff of 1.30Å for C_1 and C_3 and 1.16Å for C_2. The average number of coordination for C_1 and C_3 is four, and two for C_2. Two significant peaks show probable angles at 75 and 144 degrees with large smearing of

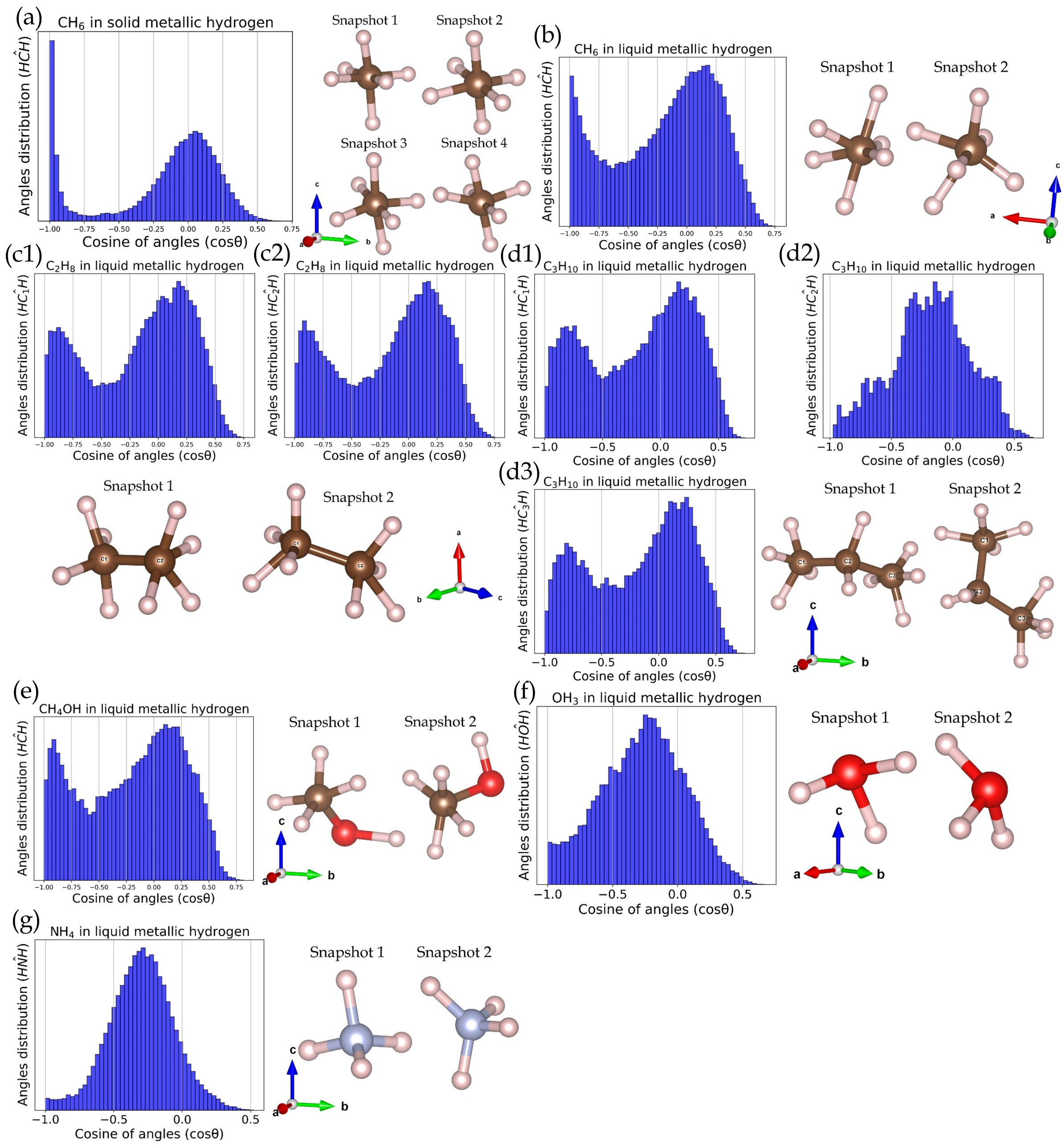

Supplementary Figure 4: Angle distributions of organic and inorganic compounds in solid metallic hydrogen and liquid metallic hydrogen between pairs of hydrogen atoms with respect to alloy atoms, such as carbon, oxygen, or nitrogen. (a) and (b) show the angle distribution of surrounding hydrogen with respect to carbon at 300 K and 600 K, respectively. (c1) and (c2) show the angle distribution of surrounding hydrogen with respect to carbon C1 and C2 at 600 K, respectively. (d1), (d2), and (d3) show the angle distribution of surrounding hydrogen with respect to carbon C1, C2, and C3 at 600 K, respectively. (e) shows the angle distribution of surrounding hydrogen with respect to carbon at 600 K. (f) shows the angle distribution of surrounding hydrogen with respect to oxygen at 600 K. (g) shows the angle distribution of surrounding hydrogen with respect to nitrogen at 600 K.

the distribution resulting from high anharmonicity of hydrogen for C_1 and C_3. The single peak shows a unique angle between two hydrogen atoms surrounding C_2 at around 104

Atomic types	Angles (degrees)
1H and 2H	87.02
1H and 3H	93.30
1H and 4H	81.97
1H and 5H	98.72
1H and 6H	178.03
2H and 3H	178.31
2H and 4H	86.88
2H and 5H	90.91
2H and 6H	91.16
3H and 4H	94.81
3H and 5H	87.40
3H and 6H	88.50
4H and 5H	177.65
4H and 6H	98.69
5H and 6H	80.54

Supplementary Table 2: shows the average angles of CH_6 in solid metallic hydrogen at 500GPa and 300K.

degrees as shown in Supplementary Figure 4.

The angle distribution of CH_4OH in liquid metallic hydrogen shows the angle distribution between pairs of hydrogen atoms with respect to carbon for each step of MD within the radius cutoff of 1.37Å where the average number of coordination is about four. Two significant peaks show probable angles at 75 and 144 degrees similar to the case of C_2H_8 and C_3H_{10} as shown in Supplementary Figure 4.

The angle distribution of OH_3 in liquid metallic hydrogen shows the angle distribution between pairs of hydrogen atoms with respect to oxygen for each step of MD within the radius cutoff of 1.32Å where the average number of coordination is about three. Single significant peak shows probable angle at 103 degrees similar to the case of C_3H_{10} as shown in Supplementary Figure 4.

The angle distribution of OH_3 in liquid metallic hydrogen shows the angle distribution between pairs of hydrogen atoms with respect to oxygen for each step of MD within the radius cutoff of 1.20Å where the average number of coordination is about four. Single significant peak shows probable angle at 110 degrees which corresponds to the angle of methane at ambient as shown in Supplementary Figure 4.

Crystal orbital Hamilton population (COHP)

To investigate the bonding characteristics between the central carbon atom and its six surrounding hydrogen atoms, we conducted a Crystal Orbital Hamilton Population (COHP) analysis. The COHP method provides a detailed understanding of bonding and antibonding interactions by quantifying the energy contributions of the orbital overlap within a chemical bond. In this study, we used conventional -COHP diagrams on the same site, where bonding states are represented as positive values (to the right) and antibonding states as negative values (to the left). The results, shown in Supplementary Figure 6, indicate that the projected Crystal Orbital Hamilton Population (-pCOHP) on the orbitals

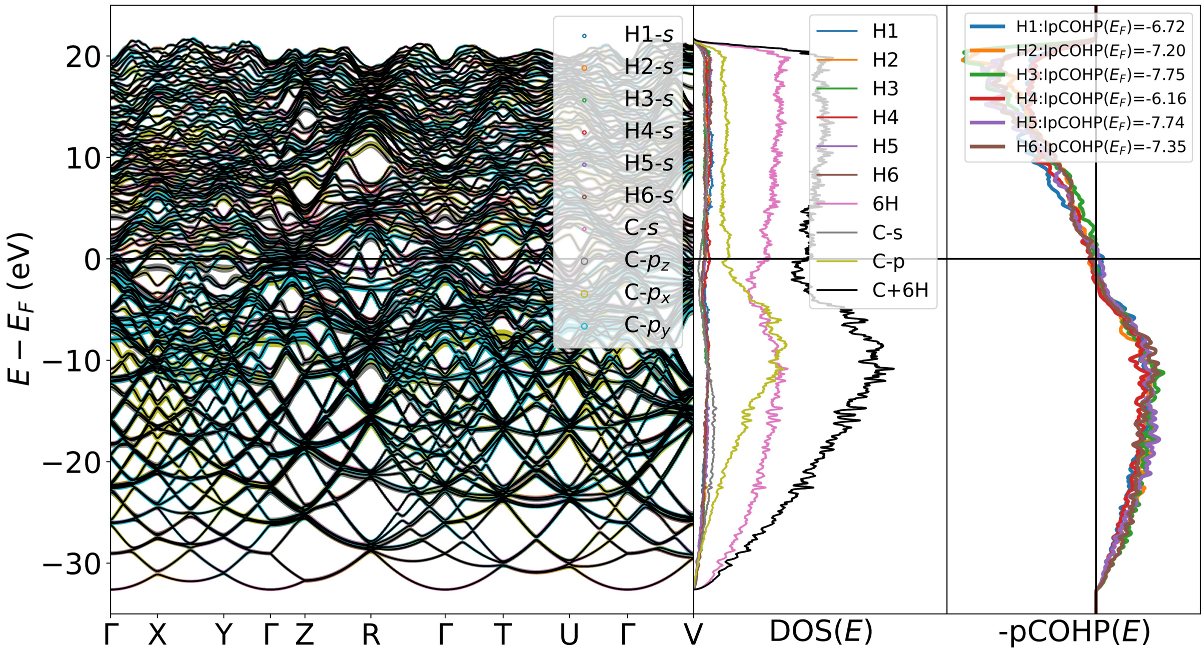

Supplementary Figure 5: show the Crystal Orbital Hamilton Population (COHP) analysis of CH_6 in solid metallic hydrogen. It shows significant covalent bonding between carbon and one of the six hydrogen atoms.

s and p for the hydrogen and carbon atoms is positive below the Fermi energy (valence electrons), which confirms the presence of bonding interactions. In particular, the results suggest orbital mixing ("hybridization") between the s and p orbitals, which is similar to the typical hybridization observed in hydrocarbon molecules under ambient conditions.

To compare with hydrogen atoms in the system that do not bond with the carbon, we calculated the -pCOHP for two additional hydrogen atoms located at distances of 1.97 Åand 3.70 Å. As shown in Supplementary Figure 6, the -pCOHP diagram indicates no bonding character, as there is no significant overlap between the wave functions of these hydrogen atoms and the central carbon atom.

To calibrate the effectiveness of COHP in this system, we carried out COHP between various pairs of atoms (Supplementary Figure 7). The CH bonds have significantly larger bonding and antibonding character than other pairs of atoms. For example, the idea that the second "shell" of hydrogens around the central carbon should be considered as a "ligand" can be ruled out by the flat COHP, indeed this has less bonding character than pairs of hydrogens far from the CH_6, or the HH neighbour pairs comprising fourfold coordinated $I4_1$/amd hydrogen.

Hypermolecules in jellium

Hypermolecules can be considered as molecular chemistry in a metallic background. In this work the metallic background was provided by metallic hydrogen, but a simpler case is to consider molecules in jellium. Jellium provides the free electron background in which the molecule is immersed, however it does not include pressure effects. The main consequence of this is that bonds can become longer with increased electron density, without incurring the free energy cost for the PV term. In high pressure solids, there is

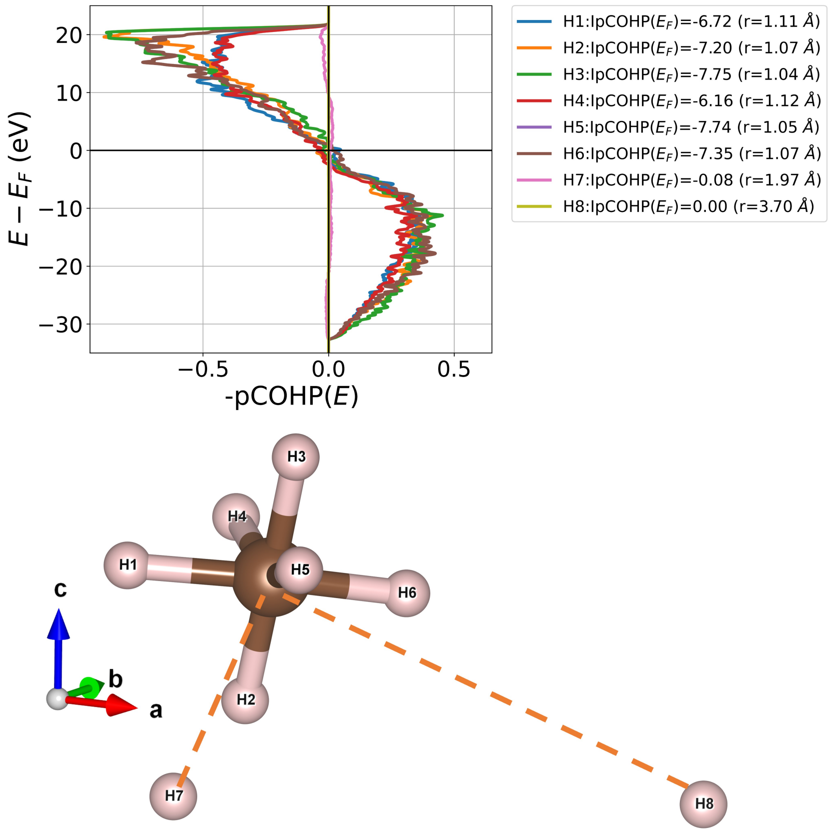

Supplementary Figure 6: show the Crystal Orbital Hamilton Population (COHP) analysis of CH_6 in solid metallic hydrogen. The analysis is extended to include two hydrogen atoms in the second and third shells. It shows no significant bonding between carbon and distant hydrogen.

typically a lengthening of the bondlength due to weakening of bonds, and a shortening due to external pressure. In the jellium model, only the former effect is present. Hence, jellium can help us to understand the electronic structure of hypermolecules, but features such as bondlengths and precise energies are of only qualitative relevance to high pressure materials.

We consider here the case of hypermethane CH_6, compared with the normal state CH_4+H_2. We calculated a large 10Å^3 box containing jellium of various densities and either a CH_6 hypermolecule or CH_4+H_2 molecules. We gradually increase the electron density and monitor the density of states to see how it affects the bonding. It is also important to note the Kohn-Sham wavefunctions follow the symmetry of the potential the Td point group for methane which gives rise to an $A1$ singlet and 3-fold degenerate $T1$ state. CH_6 with point group Oh is subject to a Jahn-Teller distortion which can significantly stabilise it (by around 3eV at $70e^-/nm^3$). This could make the critical jellium electron density as low as $40e^-/nm^3$. However this comes at the cost of enlarging the molecule making it less relevant at high pressure.

To investigate the electronic structure of hypermethane, Supplementary Figure 8 shows that the DOS of Kohn-Sham eigenstates are very clearly comprised of sharp peaks corresponding to states localised on the molecule and the characteristic square-root form of the free electron gas. The data is fitted as such, with peaks being found using the python $scipy.signal.findpeaks$ utility. As the number of electrons is increased, the bonding states

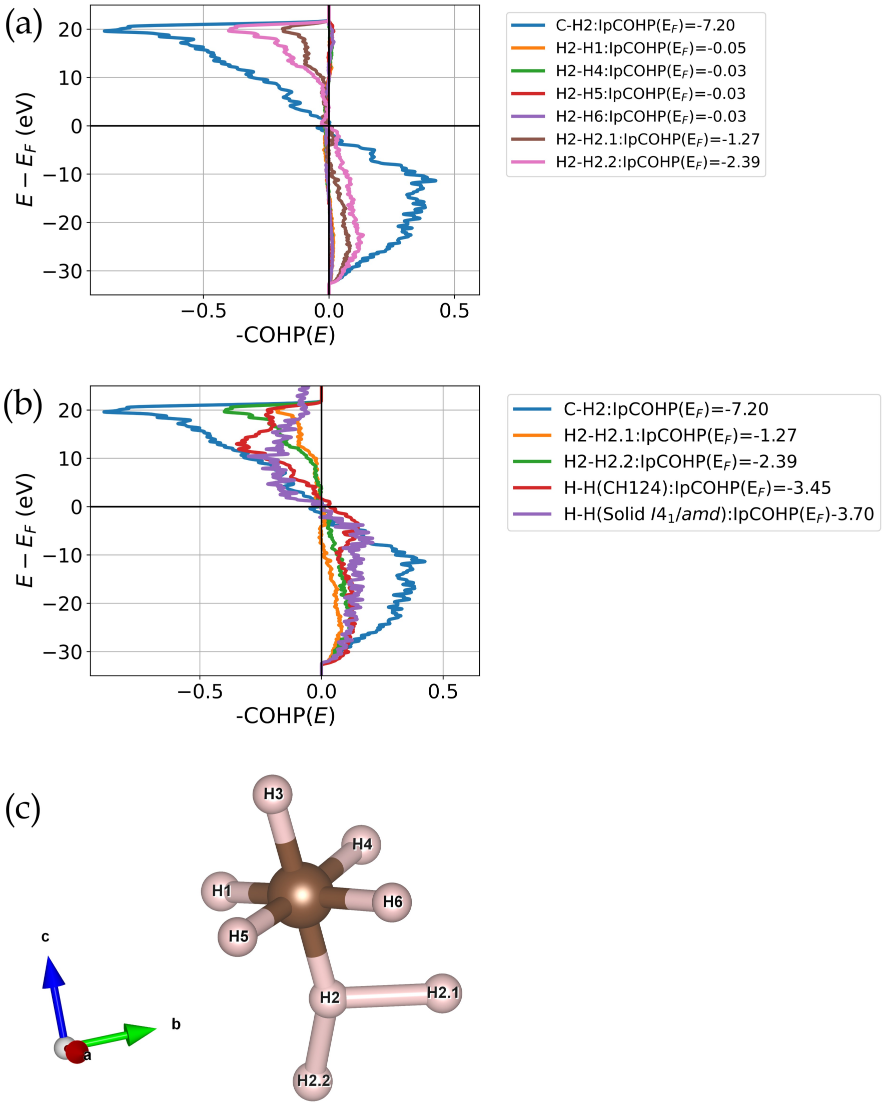

Supplementary Figure 7: COHP calculations for bonds between CH in the hypermolecule, selected H-H bonds from the hydrogens in the hypermolecule to the “ligating” hydrogens adjacent to the hypermolecule, and for hydrogen pairs in $I4_1/amd$. Figures show the COHP interactions in panels (a) and (b). Panel (a) presents the COHP interactions between the hydrogen atoms in the first shell, as illustrated in Supplementary Figure (c). Panel (b) shows the COHP interactions between hydrogen atoms in the first and second shells. In panel (b), we also compute the COHP interaction between nearby hydrogen atoms in the solid metallic background of CH_{124}, which may be zero for some pairs, as well as the COHP interaction between hydrogen atoms in solid $I4_1/amd$ metallic hydrogen, using a conventional cell with four hydrogen atoms. Panel (c) shows the visualization of carbon with its hydrogen first and outer shells.

remain approximately the same, while the free electron band extends to progressively lower energies.

A total of 10 electrons in the cell corresponds to neutral molecules with no free electrons. With 12 electrons, methane is unaffected by adding two extra electrons - they are free and delocalised. By contrast, hypermethane absorbs the electrons into localised states making a 2^- ion.

With a total of 18 electrons in the cell, 12 are localised on the CH_6 molecule. These form the six equivalent bonds which can be associated with symmetry-respecting Kohn-Sham eigenstates singlet at -16.5eV, triplet at around -9eV and further states close to the Fermi level (-2.12eV here), All energies being measure relative to the isolated electron. The CH_4 has states similar to the 2s2 and 2p6, forming the tetrahedral molecule, and the H_2 molecule also has two associated electrons.

The overlap of the free electron band and the highest four energy states in CH_6^{2-} has an important feature. The sharp band within remains approximately the same energy above the lowest free energy state - the "toe" of the square root - even as the electron density increases. This means that, unlike the bonding states, these four electron states become *increasingly stable with higher electron density.* This is the key driver of the stability of the hypermolecules.

The energetics of the competing structure are given in Supplementary Table 3. This shows that for the relaxed structure, all bonds get longer as the electron density increases. Above 70e$^-/nm$, the hydrogen molecule dissociates into two H^- ions [1], above 120e$^-/nm$ methane dissociates. If octahedral symmetry is enforced, the hypermethane is unstable at 150 e$^-/nm$, although the Jahn-Teller distorted structure is still stable. Such dissociation is a feature of the jellium model: the atoms relax to minimise their enthalpy, which they can do by increasing the size of the molecule. In a high-pressure material, such an increase in size would be penalised by the $P\Delta V$ term.

The key result is that the enthalpy difference between (CH_6+jellium) and (CH_4 + H_2 + jellium) has a crossover with increasing electron density: the hypermolecule is the stable structure is a highly metallic environment. One can understand this by considering the favorable quantum state for an electron: on the molecule or at the Fermi surface. For hydrogen, two H^- ions are more stable than an H_2 molecule because four, rather than two, electrons are removed from the high-energy states at the Fermi surface. Similarly the hypermethane becomes more stable than methane because it allows more electrons to be removed from the Fermi surface, not because the bonds are stronger.

$\rho(e^-)$ (nm^{-3})	$r_{CH}(CH_6)$Å	r_{CH} $(CH_4)\mathring{A}$	$r_{HH}(H_2)\mathring{A}$	ΔH eV
10	1.237	1.100	0.759	7.848
12	1.270	1.100	0.759	6.998
18	1.289	1.100	0.763	5.97
30	1.323	1.101	0.793	4.066
50	1.347	1.101	0.813	2.030
70	1.352	1.111	0.939	0.894
80	1.356	1.122	n/a	0.494
90	1.356	1.131	n/a	0.200
100	1.362	1.151	n/a	-0.127
120	1.386	n/a	n/a	-0.512

Supplementary Table 3: show bondlengths and energy differences. Electron density $\rho(e^-)$refers to all electrons except for the carbon 1s which is described by the ultrasoft pseudopotential. CH and HH bondlengths are given for relaxed structures, with n/a representing dissociated molecules. ΔH is the difference between hydrogen + methane and hypermethane with bondlengths fixed at the 12nm^3 values. Calculations used an 8x8x8 Monkhorst Pack k-point sampling with "precise" 424.5eV cutoff, "extreme" cutoff gives very similar ΔH and bonds of order 0.001$\mathring{A}$ shorter.

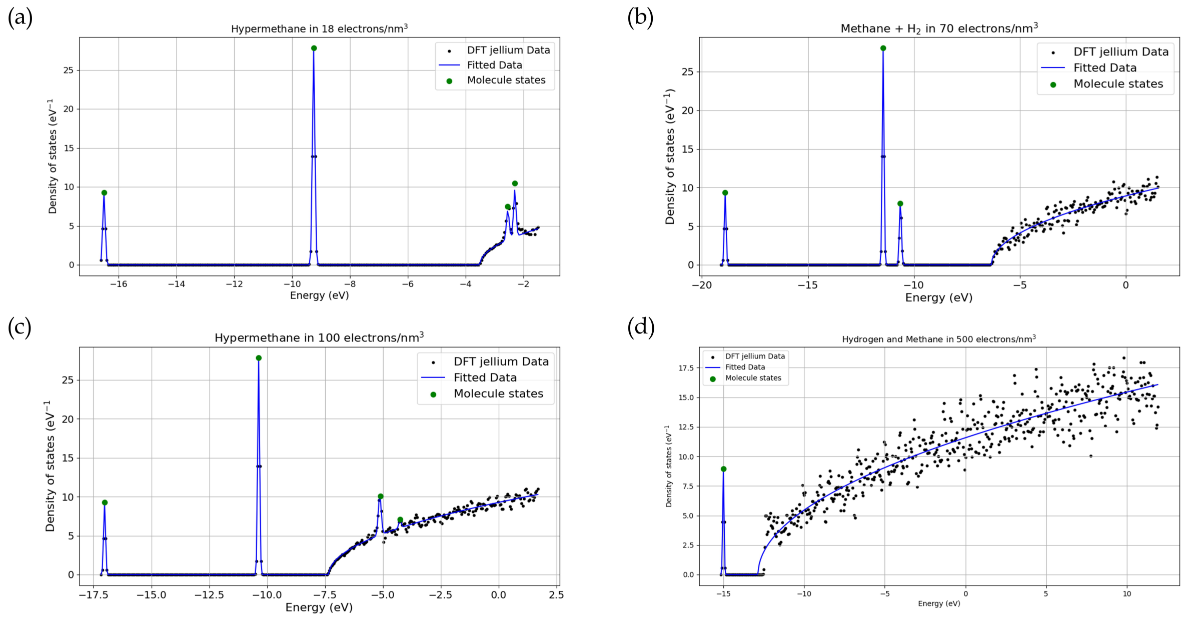

Supplementary Figure 8: show electronic density of states of molecules plus jellium in a 1nm^3 cell. Blue line shows a fit to gaussian peaks at the molecular levels, and a square root variation of the free electron gas. Data scatter comes from finite k-point sampling in the DFT calculation. a) hypermethane CH_6 with 18e, indicated peaks contain 2, 6, 2 and 2 localised electrons b) hypermethane CH_4H_2, c) hypermethane CH_6, at higher electron density. d) CH_6 at 100e

Phonon density of states of metallic hydrogen and carbon of diamond

Supplementary Figure 9 shows a comparison of the phonon density of states obtained from DFPT and BOMD calculations, demonstrating the reliability of the BOMD results with respect to the DFPT method.

Finite size effect

We have checked for finite size effects. Our initial analysis is based on partial RDFs of CH and OH separations, as shown in Supplementary Figure 10 (left). This shows that simulations of around one hundred or one thousand atoms gives the same hypermolecule formation, both number of bonds and bondlength in Supplementary Figure 10 (right). We observe liquid structure peaks which extend beyond the small unit cell size, however, within a thousand-atom simulation, this oscillating structure has decayed away exponentially (Supplementary Figure 10). Therefore, we are confident that the results from our hundred-atom simulations, although not reaching the screen length, do give a good description of the hypermolecules.

Mulliken charges

In this section, we show Mulliken charge of molecular compounds in solid and liquid metallic hydrogen. Supplementary Table 4 shows Mulliken charge of CH_6 in solid metallic

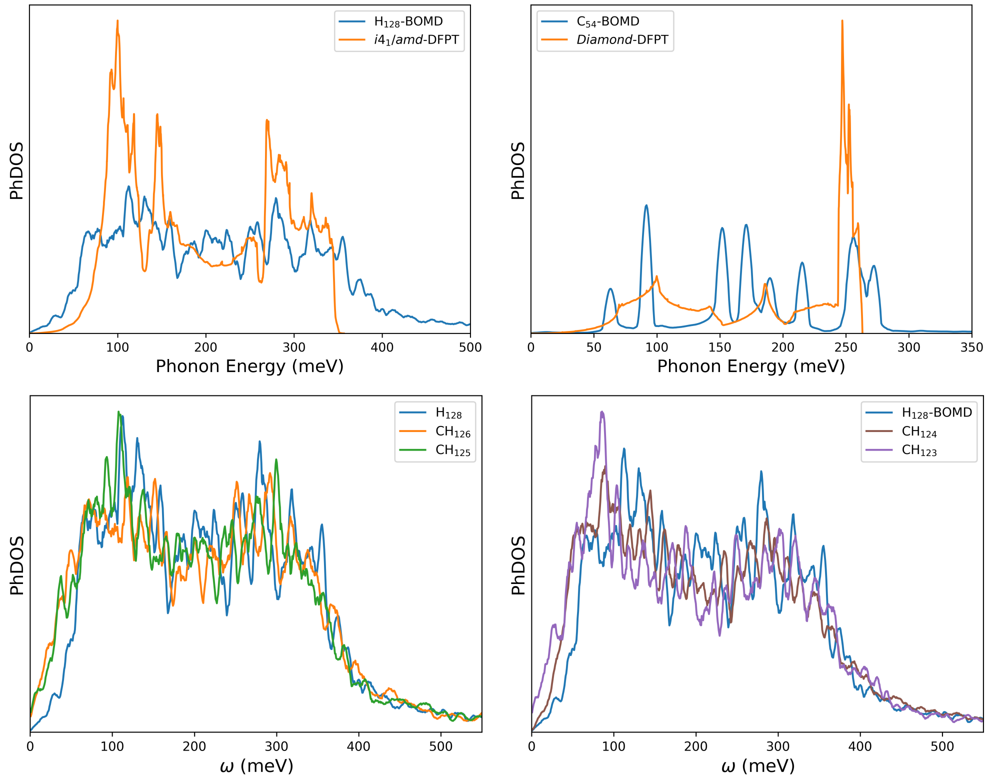

Supplementary Figure 9: show the phonon density of states compared between DFPT and BOMD calculations. (Upper left): The phonon density of states of $I4_1/amd$ at 500 GPa (DFPT: orange, BOMD: blue). (Upper right): The phonon density of states of carbon at 500 GPa with the diamond structure (DFPT: orange, BOMD: blue). (Lower left): The phonon density of states from BOMD of $I4_1/amd$ (blue), CH_{126} (orange), and CH_{125} (green). (Lower right): The phonon density of states from BOMD of $I4_1/amd$ (blue), CH_{124} (orange), and CH_{123} (green).

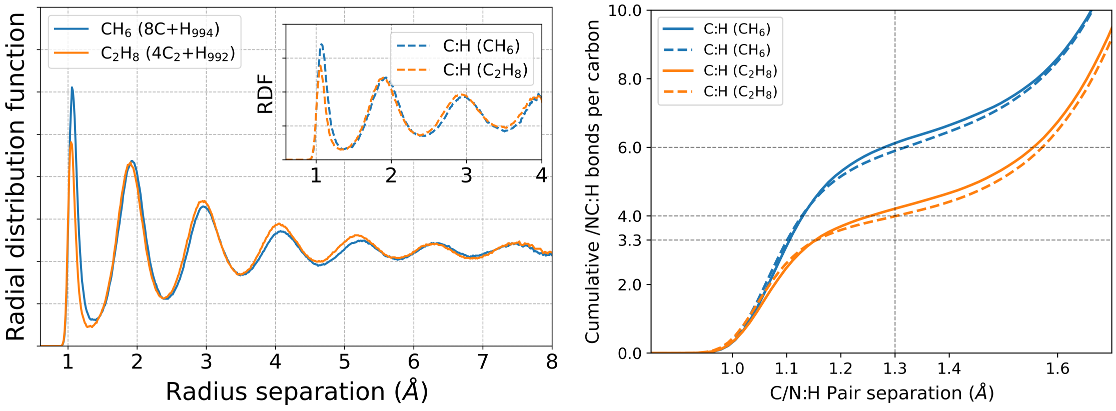

Supplementary Figure 10: show radial function distribution, and cumulative number of bonds per carbon of simulations of around one hundred or one thousand atoms.

hydrogen from four snapshots of CH_6 in Supplementary Figure 4.

For solid metallic hydrogen, we have the same hydrogen for the whole MD simulation. However, for liquid metallic hydrogen, hydrogens show in Supplementary Table 5 are different for the two different snapshots. We refer the label of carbon in Supplementary

CH_6 in solid hydrogen	Mulliken 1 (-e)	Mulliken 2 (-e)	Mulliken 3 (-e)	Mulliken 4 (-e)
C	-0.358	-0.371	-0.382	-0.358
H	0.068	0.091	0.062	0.077
H	0.083	0.071	0.065	0.072
H	0.058	0.058	0.056	0.062
H	0.067	0.079	0.094	0.069
H	0.059	0.082	0.088	0.073
H	0.057	0.050	0.084	0.065

Supplementary Table 4: Table shows the Mulliken charge of from different snapshots of CH_6 in solid metallic hydrogen at 500GPa and 300K.

Table 6, Supplementary Table 7 to carbon atoms in Supplementary Figure 4. The different snapshots of Mulliken charges (different Mulliken 1, 2, 3 and 4) correspond to different snapshots in molecular compounds showing in Supplementary Figure 4 for CH_6 in solid metallic hydrogen, CH_6, C_2H_8, C_3H_{10}, CH_4OH, OH_3, and NH_4. From Mulliken charge of CH_6 in solid metallic hydrogen as shown in Supplementary Table 4 which is similar to Mulliken charge in the case of CH_6 in liquid metallic hydrogen as shown in Supplementary Table 5, it shows that all six bonds are equivalent to each other.

CH_6 in liquid hydrogen	Mulliken 1 (-e)	Mulliken 2 (-e)
C	-0.367	-0.365
H	0.083	0.050
H	0.062	0.033
H	0.059	0.042
H	0.062	0.070
H	0.105	0.070
H	0.053	0.062

Supplementary Table 5: Table shows the Mulliken charge of from different snapshots of CH_6 in liquid metallic hydrogen background at 500GPa, 600K.

For C_2H_8 in liquid metallic hydrogen as shown in Supplementary Table 6, Mulliken charges show that carbons (C1 and C2) atoms have the same Mulliken properties so do as four hydrogen atoms. Therefore, it shows that these bonds are equivalent to each other.

For C_3H_{10} in liquid metallic hydrogen, we also have equivalent bonds of hydrogen atoms among C1, C2 and C3 as a result of similar pattern of Mulliken charges between each carbon atom and their hydrogen atoms as shown in Supplementary Table 7.

For CH_4OH in liquid metallic hydrogen, Mulliken charges as shown in Supplementary Table 8 suggests bonds between the four hydrogen atoms and the carbon atom are equivalent. The larger Mulliken charge in oxygen's hydrogen also suggest strong charge transfer, stronger bond between the hydrogen and oxygen.

For OH_3 and NH_4 in liquid metallic hydrogen as shown in Supplementary Table 9 and Supplementary Table 10, it shows similar Mulliken charge between hydrogens and oxygen and nitrogen with large Mulliken charges corresonding to stronger bonds than between those hydrogens and carbons. Therefore, the bonds between oxygen or nitrogen with their hydrogen atoms are equivalent.

In general, the Mulliken charge of all previous supplementary tables suggests that the bonding between alloys and their corresponding hydrogen atoms is equivalent, which we

C_2H_8 in liquid hydrogen	Mulliken 1 (-e)	Mulliken 2 (-e)
C1	-0.291	-0.248
H	0.059	0.036
H	0.084	0.070
H	0.083	0.070
H	0.059	0.059
C2	-0.278	-0.272
H	0.084	0.058
H	0.077	0.065
H	0.056	0.051
H	0.090	0.073

Supplementary Table 6: Table shows the Mulliken charge of from different snapshots of C_2H_8 in liquid metallic hydrogen background at 500GPa, 600K.

C_3H_{10} in liquid hydrogen	Mulliken 1 (-e)	Mulliken 2 (-e)
C1	-0.321	-0.295
H	0.091	0.073
H	0.097	0.059
H	0.076	0.077
H	0.107	0.071
C2	-0.173	-0.146
H	0.047	0.047
H	0.059	0.049
C3	-0.263	-0.277
H	0.050	0.066
H	0.076	0.067
H	0.105	0.071
H	0.068	0.048

Supplementary Table 7: Table shows the Mulliken charge of from different snapshots of C_3H_10 in liquid metallic hydrogen background at 500GPa, 600K.

call covalent bonds in our investigation.

CH_4OH in liquid hydrogen	Mulliken 1 (-e)	Mulliken 2 (-e)
C	-0.069	-0.081
H	0.065	0.063
H	0.073	0.077
H	0.053	0.066
H	0.050	0.062
O	-0.438	-0.442
H	0.261	0.163

Supplementary Table 8: Table shows the Mulliken charge of from different snapshots of CH_4OH in liquid metallic hydrogen background at 500GPa, 600K.

OH_3 in liquid hydrogen	Mulliken 1 (-e)	Mulliken 2 (-e)
O	-0.519	-0.469
H	0.144	0.201
H	0.213	0.251
H	0.221	0.232

Supplementary Table 9: Table shows the Mulliken charge of from different snapshots of OH_3 in liquid metallic hydrogen background at 500GPa, 600K.

NH_4 in liquid hydrogen	Mulliken 1 (-e)	Mulliken 2 (-e)
N	-0.415	-0.428
H	0.161	0.189
H	0.174	0.168
H	0.169	0.133
H	0.160	0.134

Supplementary Table 10: Table shows the Mulliken charge of from different snapshots of NH_4 in liquid metallic hydrogen background at 500GPa, 600K.

Supplementary references

[1] Marqués M, Peña-Alvarez M, Martínez-Canales M and Ackland G J 2023 *The Journal of Physical Chemistry C* **127** 15523–15532
